# Identification and quantification of chimeric sequencing reads in a highly multiplexed RAD‐seq protocol

**DOI:** 10.1111/1755-0998.13661

**Published:** 2022-06-27

**Authors:** Maria Luisa Martin Cerezo, Rohan Raval, Bernardo de Haro Reyes, Marek Kucka, Frank Yingguang Chan, Jarosław Bryk

**Affiliations:** ^1^ Department of Biological and Geographical Sciences, School of Applied Sciences University of Huddersfield Huddersfield UK; ^2^ IFM Biology Linköping University Linköping Sweden; ^3^ Friedrich Miescher Laboratory of the Max Planck Society Tübingen Germany

**Keywords:** adapters, barcodes, chimeras, index hopping, quaddRAD, RAD‐seq, read misassignment

## Abstract

Highly multiplexed approaches have become common in genomic studies. They have improved the cost‐effectiveness of genotyping hundreds of individuals using combinatorially barcoded adapters. These strategies, however, can potentially misassigned reads to incorrect samples. Here, we used a modified quaddRAD protocol to analyse the occurrence of index hopping and PCR chimeras in a series of experiments with up to 100 multiplexed samples per sequencing lane (639 samples in total). We created two types of sequencing libraries: four libraries of type A, where PCRs were run on individual samples before multiplexing, and three libraries of type B, where PCRs were run on pooled samples. We used fixed pairs of inner barcodes to identify chimeric reads. Type B libraries show a higher percentage of misassigned reads (1.15%) than type A libraries (0.65%). We also quantify the commonly undetectable chimeric sequences that occur whenever multiplexed groups of samples with different outer barcodes are sequenced together on a single flow cell. Our results suggest that these types of chimeric sequences represent up to 1.56% and 1.29% of reads in type A and B libraries, respectively. We also show that increasing the number of mismatches allowed for barcode rescue to above 2 dramatically increases the number of recovered chimeric reads. We provide recommendations for developing highly multiplexed RAD‐seq protocols and analysing the resulting data to minimize the generation of chimeric sequences, allowing their quantification and a finer control on the number of PCR cycles necessary to generate enough input DNA for library preparation.

## 1 INTRODUCTION

The development of high‐throughput sequencing and reduced‐representation approaches, such as restriction site‐associated DNA sequencing (RAD‐seq), has dramatically reduced the cost of generating vast amounts of sequencing data. RAD‐seq (Baird et al., 2008) and its many variants, including but not limited to: ddRAD‐seq (Peterson et al., 2012), quaddRAD‐seq (Franchini et al., 2017) and adapterama (Bayona‐Vásquez et al., 2019; Glenn et al., 2019), have been used in studies of phylogenetics (Lecaudey et al., 2018; Massatti et al., 2016; Near et al., 2018), phylogeography (Jeffries et al., 2016), association mapping (Nadeau et al., 2014), introgression (Hohenlohe et al., 2013), population structure and genetic diversity (Gao et al., 2017; Leone et al., 2019; Martin Cerezo et al., 2020; Rodríguez‐Ezpeleta et al., 2016).

Many high‐throughput methods rely on multiplexing, inclusion of unique identifying sequences in the adapters of each sample (barcodes or indices), pooling of samples and subsequently sequencing pools on a single sequencing lane. This approach has now become common practice, and single (Poland & Rife, 2012), dual (Glenn et al., 2019; Peterson et al., 2012) and quadruple (Bayona‐Vásquez et al., 2019; Franchini et al., 2017) barcodes have been developed. While these approaches greatly reduce the costs of sequencing, they can also increase the number of misassigned reads (MacConaill et al., 2018), where sequences from one sample are incorrectly assigned to another due to sequencing errors, nucleotide misincorporations and contamination of adapters during synthesis or library preparation (van der Valk et al., 2020), among others.

PCR chimeras are reads composed of two different parental sequences, and are one source of read misidentification (Fonseca et al., 2012) and are caused by spontaneous dissociation of polymerases from the template molecules during which amplification can occur. It can occur due to low processivity, secondary structures of DNA or incomplete extension of DNA during the PCR (Smyth et al., 2010), and can lead to the formation of incomplete sequences. These fragments can act as primers during subsequent amplification cycles and produce artificial PCR products containing fragments of sequences containing barcodes from two different samples. Index hopping, in contrast, is caused by free‐floating primers resulting from insufficient DNA purification, erroneous size selection of the library or fragmentation during improper storage; these primers can prime template DNA molecules on a sequencing flow cell prior to exclusion amplification (van der Valk et al., 2020). The presence of both chimeric and index‐hopped sequences can lead to inflated measures of diversity, and bias population genetic parameters in downstream analyses (Smyth et al., 2010; van der Valk et al., 2020).

Additionally, chimeras can occur in any RAD‐seq protocol whenever a single flow cell is filled with groups of samples that were processed independently but which share inner barcodes. In such cases, the chimeric sequences are impossible to differentiate from genuine samples during downstream analysis. Only when unique combinations of both inner and outer barcoded adapters are used, such chimeric reads can be identified, quantified and eliminated during analysis. The quaddRAD protocol, due to its split four‐part adapters, allows for the identification of chimeric sequences (Franchini et al., 2017). While PCR‐free protocols for library preparation can alleviate the problem of read misassignment, studies have found considerable read misassignment in these libraries (Costello et al., 2018). Furthermore, they require a large amount of high‐quality DNA, which is often not available.

The incidence of index hopping during cluster generation of the Illumina HiSeq X and NovaSeq platforms has been reported as <1% (van der Valk et al., 2020). However, the still widely used platforms utilizing exclusion amplification (ExAmp) cluster generation such as the HiSeq 3000/4000 have reported misassignment rates up to 10% (Sinha et al., 2017), and up to 30% in PCRs (Wang & Wang, 1996). We note, however, that published analyses of chimeric sequences were obtained on relatively few samples, and considered formation of chimeric sequences only during cluster generation or only during library preparation.

Here, we quantify the prevalence of chimeric sequences in two large‐scale, highly multiplexed experiments (86 to 100 samples multiplexed per lane of sequencing, total number of samples = 639). We assessed the contribution of both PCR amplification and sequencing to the generation of chimeric sequences, by preparing two types of libraries: type A, where adapter ligation and PCR amplification were carried out on each sample individually, and type B, where samples were pooled before amplification and addition of outer adapters, respectively. Our design also allows for identification of chimeric sequences that are otherwise undetectable: sequences formed between groups of samples that share some combinations of inner adapters, but those were processed in different multiplexed groups. Overall, we identify and quantify four types of chimeric sequences. Based on our findings, we provide recommendations for adapter design and data analysis to minimize the number of misassigned reads.

## 2 MATERIALS AND METHODS

### 2.1 Adapter design

Adapters were designed following the quaddRAD protocol (Franchini et al., 2017). Restriction enzyme overhangs were modified for SbfI and MseI. 8‐bp‐long barcodes were designed using EDITTAG (Faircloth & Glenn, 2012), with a minimum Levenshtein distance of 4 nucleotides, GC content of 40–60% and avoiding sequences that were self‐complementary and containing more than two adjacent, identical bases. From 102 tags suggested by EDITTAG, sequences that reconstructed SbfI and MseI restriction sites were removed manually. 18 tags were selected for the inner adapters and 8 for the outer adapters, giving a total of 144 possible combinations.

**FIGURE 1:**
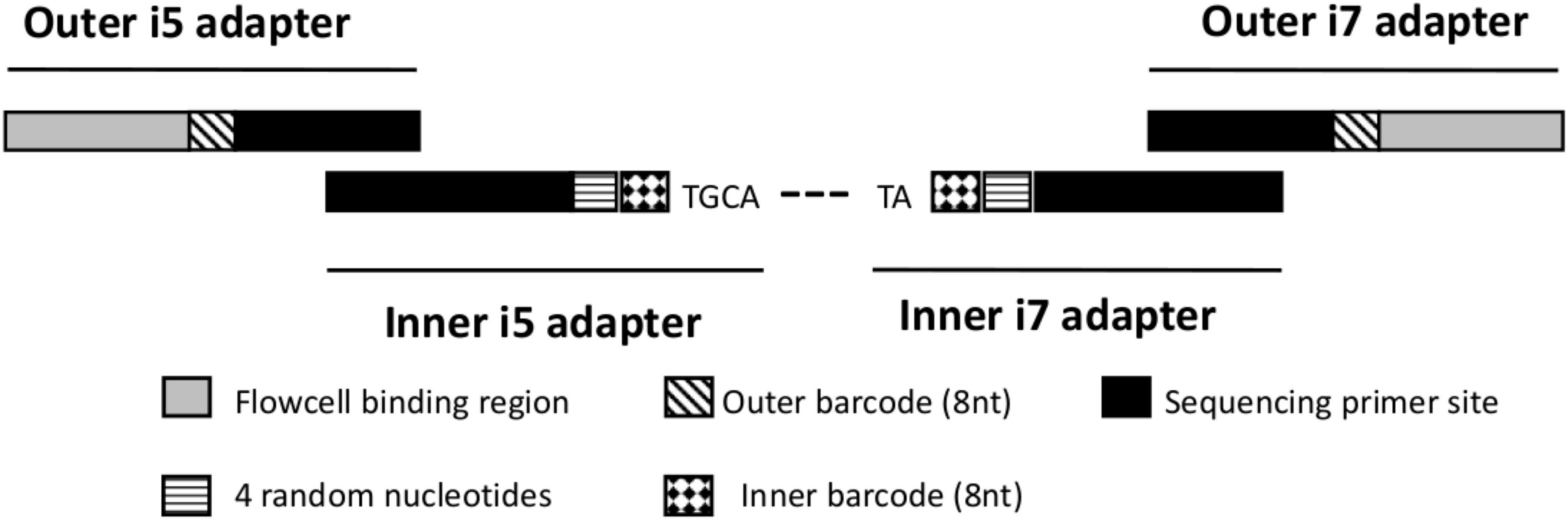
Elements of adapter sequences. Modified from Franchini et al. (2017)

Four random nucleotides (5’‐VBBN‐3′) were also incorporated into the inner adapters to allow in silico identification of PCR duplicates (Figure 1). Inner adapters were used in fixed pairs while outer adapters have been used combinatorially. A complete list of adapter sequences can be found in Tables S1–S3.

### 2.2 Library preparation

DNA from 459 *Apodemus flavicollis* and 180 *Apodemus sylvaticus* tissue samples was extracted following Martin Cerezo et al. (2020). Seven libraries were prepared following a modified version of the quaddRAD Franchini et al. (2017) protocol.

Four libraries (*n* = 164 *A. flavicollis* and 180 *A. sylvaticus*, 86 samples per library), henceforth called type A, were prepared with each sample individually amplified to allow for the quantification of sequencing chimeras or index‐hopped reads only. The three remaining libraries (*n* = 295 *A. flavicollis* samples including 96, 99 and 100 samples per library), henceforth called type B, were multiplexed following restriction digestion and ligation of barcoded inner adapters before they were amplified as a pool. This allowed for the quantification of the total number of chimeric sequences, which originated both during PCR amplification and during sequencing, as well as during PCR amplification alone.

#### 2.2.1 Type A libraries: Individual PCRs

Inner adapters were prepared by annealing each single‐stranded oligonucleotide with its complementary strand. 5 μl of each bottom and top strand at 100 μM was mixed with 40 μl of annealing buffer (50 mM NaCl, 10 mM Tris‐Cl, pH 8.0), heated to 98°C for 2.5 min and cooled at a rate of 1°C per minute down to 15°C. Once prepared, the adapters were kept at −20°C and used within 2 weeks.

60 ng of genomic DNA was then digested and ligated to the inner adapters in a single‐step 40 μl reaction containing 4 μl 1× CutSmart buffer, 1.5 μl *Mse1* (10 U/μl), 0.75 μl *Sbf1* (20 U/μl), 4 μl ATP (10 mM), 1 μl T4 DNA ligase (400 U/μl), 0.75 μl of each quaddRAD_i5n and quaddRAD_i7n inner adapters (10 μM), and ddH_2_ O to 40 μl and incubated for 3 h at 30 °C in a thermocycler. The reaction was stopped with 10 μl of 50 mM EDTA. Samples were purified and double‐size‐selected using 0.4× and 0.8× Sera‐Mag SpeedBeads solution (GE Lifesciences, Marlborough, MA, USA) containing 10 mM Tris base, 1 mM EDTA, 2.5 M NaCl, 20% PEG 8000 and 0.05% Tween‐20 (pH 8.0), and eluted in 30 μl 10 mM Tris–HCl.

To introduce the outer barcoded adapters, an indexing PCR was carried out in a 50 μl reaction containing 4 μl of each i5 and i7 primers (5 mM), 1 μl of dNTPs (10 mM), 10.5 μl of purified water, 10 μl of 5x Q5‐HF Buffer, 0.5 μl of Q5‐HF DNA Polymerase (New England Biolabs, Frankfurt am Main, Germany) and 20 μl of template DNA. After an initial denaturation step of 30 s at 98°C, the PCR was carried out in 14 cycles (15 s at 98°C, 30 s at 67°C and 60 s at 72°C) and a final elongation at 72°C for 2 minutes. Purification was performed using 0.8x Sera‐Mag SpeedBeads solution (GE Lifesciences, Marlborough, MA, USA), and DNA was eluted in 22 μl Tris‐HCl (10 mM).

Samples were multiplexed by combining 10 ng of each sample in Plates 1 and 4 and 20 ng of each sample in Plates 2 and 3. Libraries were then size‐selected to 300–600 bp using BluePippin (Sage Science, Beverley, MA, USA) and sequenced on Illumina HiSeq 3000 (Illumina Inc., San Diego, CA, USA).

#### 2.2.2 Type B libraries: Multiplexed PCR

Digestion and ligation reactions were performed as described for type A libraries, except with an initial input of 100 ng of genomic DNA. Samples were then purified using 0.8× SPRI Sera‐Mag SpeedBeads solution, eluted in 30 μl of Tris–HCl (10 mM) and subsequently equimolarly pooled according to inner barcode combinations prior to PCR amplification.

An indexing PCR was carried out to introduce the outer barcoded adapters to each pool of digested DNA and enrich the libraries in a 100 μl reaction containing 8 μl dNTP mix (2.5 mM), 20 μl 5× Q5‐HF buffer, 4 μl quaddRAD‐i5nn primer (10 μM), 4 μl quaddRAD‐i7nn primer (10 μM), 1 μl Q5 high‐fidelity DNA polymerase (2 U/μl), 50 ng of DNA (restricted, ligated and pooled) and ddH_2_ O to 100 μl. Reaction conditions were as described for type A libraries. Each PCR was again purified using 0.8× SPRI Sera‐Mag SpeedBeads solution and eluted in 50 μl of Tris‐HCl (10 mM). 100 ng of each enriched library was then pooled again and size‐selected to 300–600 bp using Blue Pippin (Sage Science, Beverly, MA, USA) and sequenced on Illumina HiSeq 3000 (Illumina Inc., San Diego, CA, USA).

### 2.3 Clone removal

Sequences were demultiplexed based on the outer barcodes by the sequencing centre (Genome Centre at the Max Planck Institute for Developmental Biology, Tübingen, Germany). PCR duplicates were identified and removed from each library using the clone_filter programme from Stacks 2.41 (Catchen et al., 2011) and the random nucleotide tags in the inner adapters. Sequences were then demultiplexed based on the inner barcodes, quality‐filtered and truncated to 136 bp with process_radtags, also from Stacks, removing reads with uncalled bases, low‐quality scores, and reads that were marked by Illumina's chastity/purity filter as failing and allowing for barcode and RAD‐tags rescue. process_radtags was run 5 times, changing the number of mismatches allowed for barcode rescue from 0 to 4 at each iteration. Samples were demultiplexed using not only combinations of barcodes used for library preparation but also all the possible combinations of inner barcodes, allowing for the quantification of chimeric sequences. The number of retained reads for each barcode combination and for each one of the process_radtags runs was recovered from the log files generated by process_radtags.

### 2.4 Multiplexed groups

Multiplexed groups are defined as a set of samples that share outer adapters. Typically, several of such groups are prepared in parallel, pooled and then sequenced on a single lane of a sequencing machine. Each one of the four type A libraries included 8 multiplexed groups of 9 individuals each, 1 multiplexed group of 8 individuals and 1 multiplexed group with 6 individuals. Type B libraries had different multiplexed schemes per library. The libraries contained 8 multiplexed groups of 9 individuals and 3 multiplexed groups of 8 individuals. Library type B‐1 also contained an additional multiplexed group with 4 individuals, while library type B‐2 contained a multiplexed group with 3 individuals.

### 2.5 Identification of chimeric sequences

**FIGURE 2:**
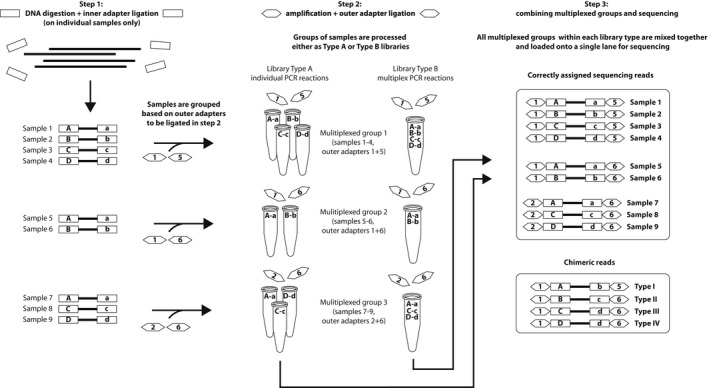
Summary of the experimental design and the types of chimeric reads. Inner adapters are represented by letters and rectangles while outer adapters are represented by numbers and hexagons. Combinations of the same uppercase and lowercase letters represent the unique combinations of inner barcoded adapters used in the protocol. Any other combination of letters represents chimeric combinations of adapters. Multiplexed groups are groups of samples processed together in the protocol

Based on the combination of inner and outer barcodes used, we divided the identified chimeric sequences into four types. A summary of the experimental design and the types of chimeric reads are shown in Figure 2.

Type I chimeras are molecules that contain unused combinations of inner barcodes, both of which were used in the same multiplexed group. For example, inner barcodes ‘A’, ‘a’, ‘B’ and ‘b’ were present in multiplexed group 1, but not in a combination ‘Ab’.

Chimeras of types II–IV form between sequences from different multiplexed groups and are only detectable if multiplexed groups contain different numbers of combinations of inner barcodes. For example, multiplexed group 1 contains 4 combinations of inner barcodes (Aa, Bb, Cc, and Dd) but multiplexed group 2 contains only 2 (Aa, Bb). These chimeras are detectable when samples are processed in several unequally sized groups, but the same set of inner barcodes is used across all groups.

Type II chimeras are reads containing one inner barcode that was used in a different multiplexed group. For example, the combination of inner barcodes ‘Bc’ in multiplexed group 2 is a chimera type II since this multiplexed group contains barcode ‘B’ but not barcode ‘c’.

Type III chimeras are reads containing a *chimeric* combination of inner barcodes, neither of which was used in their multiplexed group. An example of these type of chimeras is the combination of inner barcodes ‘Cd’ in multiplexed group 2, since this multiplexed group does contain neither barcodes ‘C’ nor ‘d’. Barcode ‘C’ was used in multiplexed group 1 and barcode ‘d’ in multiplexed group 3.

Finally, type IV chimeras are reads containing a correct combination of inner barcodes that were used in other multiplexed groups but not in the group where they were detected. The combination ‘Dd’ in multiplexed group 2 is one of these chimeras since multiplexed group 2 does not contain barcodes ‘D’ nor ‘d’, but this combination of barcodes was used in multiplexed groups 1 and 3. In our protocol, it was only possible to detect type IV chimeras in 19 of 75 multiplexed groups as all other groups were equally sized.

### 2.6 Counts of sequences with chimeric adapters

For each multiplexed group, unused combinations of barcodes were used to detect chimeric sequences and were quantified as a percentage of total sequences within the multiplexed group. Each of the multiplexed groups included between 2 and 9 samples. We calculated percentage of chimeric sequences in relation to mismatches allowed for barcode rescue and the library type. This allowed comparison of the proportion of chimeras when different numbers of mismatches were allowed, as well as comparison of the results within and between each library type.

To compare the relative abundance of the different types of chimeras, the percentage of chimeric sequences of each type was calculated relative to the number of reads per plate sequenced. Similarly, the percentage of chimeric sequences was also calculated individually for each possible chimeric combination of barcodes.

Type IV chimeras can only be identified in very specific multiplexing schemes and barcode combinations, but it is worth emphasizing that they are being generated in all multiplexed groups, whether or not the experimental protocol enables their detection. We can directly quantify a fraction of type IV chimeras in 19 of our 75 multiplexed groups, but we expect that in other multiplexed groups or combinations of barcodes, type IV chimeras represent a similar percentage of misassigned reads. We estimated the number of type IV chimeras, including their generation between all possible combinations of adapters that could produce them, with the following formula:

$$\mathrm{est}\_\text{chimIV}=\frac{\frac{\mathrm{obs}\_\text{chimIV}}{\text{total}\_\mathrm{mg}\_\text{reads}}\times \text{total}\_\text{reads}}{\frac{\mathrm{pos}\_\text{chimIV}}{\text{total}\_\text{chimIV}}}$$

where obs_chimIV, number of observed type IV chimeras; total_mg_readsm number of reads in multiplexed groups with observed type IV chimeras; pos_chimIV, total number of cases that could produce type IV chimeras; total_chimIV, number of cases where type IV chimeras were identified; total_reads, total number of reads.

Calculations were performed only considering 0 mismatches for barcode rescue.

## 3 RESULTS

### 3.1 Multiplex PCR increases the proportion of sequences with chimeric adapters

**FIGURE 3:**
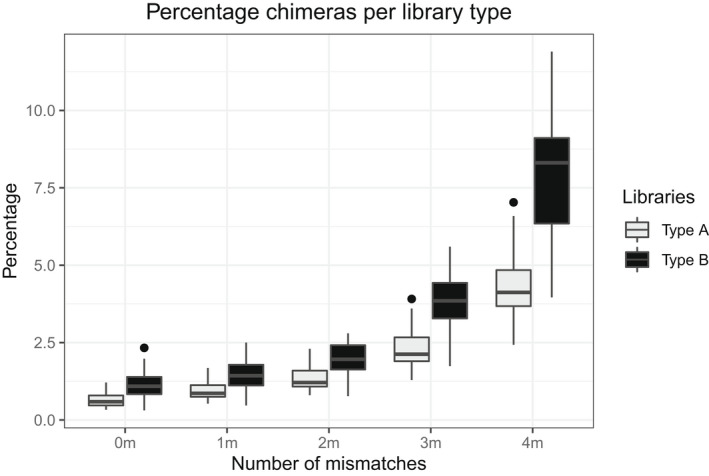
Percentage of chimeric sequences in type A (grey; PCR on individual samples: includes only sequencing chimeras) and type B libraries (black, PCR on multiplexed groups of samples, includes PCR and sequencing chimeras) for each level of barcode rescue

Type A libraries, where indexing PCRs were conducted on each sample independently, consistently produced fewer chimeric sequences, as a percentage of total sequences, than type B libraries, at the same number of mismatches during barcode rescue (Figure 3). Overall, demultiplexing with perfect barcodes showed a median of 0.59% (max = 1.20%, min = 0.33%, mean = 0.65%) and 1.09% (max = 2.33%, min = 0.31%, mean = 1.15%) chimeric sequences for type A and type B libraries, respectively.

Increasing the number of mismatches for barcode rescue from zero to four also increased the percentage of chimeric sequences detected in both library types to a median of 4.12% (max = 7.03, min = 2.43, mean = 4.37%) for type A and 8.31% (max = 11.90, min = 3.96, mean = 7.85%) for type B, as a greater number of reads were retained by process_radtags. In all cases, differences between type A and type B libraries were significant (Figure 3, Mann–Whitney U test, *p* < .001).

### 3.2 Differences in percentage of chimeric sequences within type A or type B libraries are smaller than between the two libraries

**FIGURE 4:**
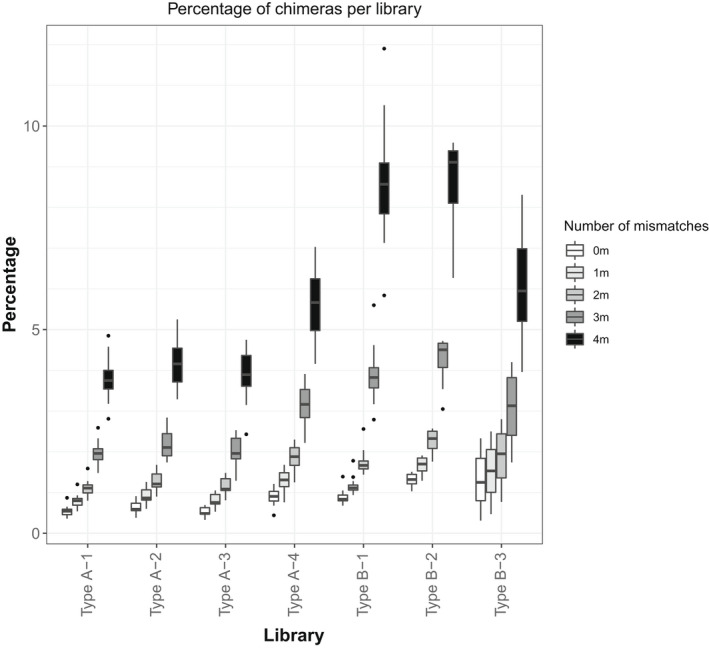
Percentage of chimeric sequences in independently prepared libraries of type A and type B for each level of barcode rescue

Percentage of chimeric sequences within independently prepared libraries of the same type (four libraries of type A and three of type B) were more similar to one another than between libraries of different types (Figure 4). The only significant differences between libraries of the same type were between type A‐4 vs type A‐1 and type A‐4 vs type A‐3 (H_3_ = 13.3, *p* = .004, post hoc Dunn’s test). No other significant differences were detected between any other combination of libraries.

### 3.3 The proportion of chimeric sequences increases with the number of mismatches allowed in barcode rescue

Allowing mismatches for barcode rescue enables recovery of sequences with uncalled or erroneous base calls in the barcode sequence. Therefore, any increment in the number of mismatches will increase the number of reads retained after demultiplexing with process_radtags. It will also increase the proportion of chimeric sequences.

**FIGURE 5:**
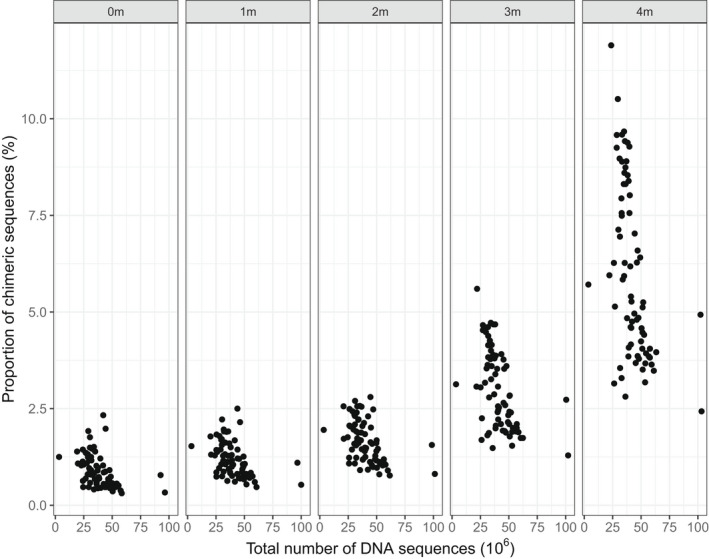
Percentage of chimeric sequences in total number of DNA sequences recovered from both library types for each level of barcode rescue

The overall proportion of such sequences increased significantly with each increment in the number of mismatches allowed for barcode rescue, from 0 to 4 (Kruskal–Wallis test: *H*_4_ = 287, *p* < .001). The closer the number of mismatches is to the distance between barcodes (in our case, 4 nucleotides), the larger the increase in the proportion of chimeric sequences (Figure 5).

**FIGURE 6:**
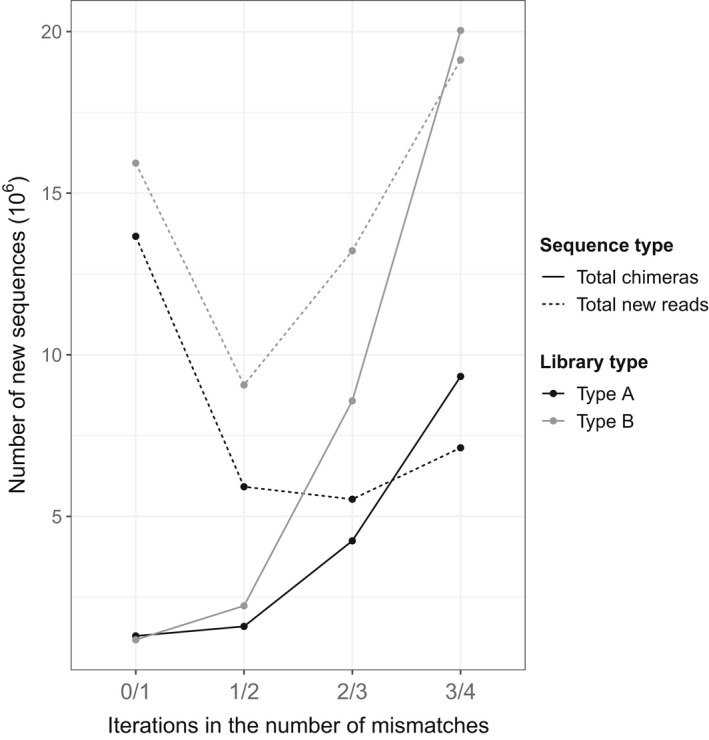
Number of new sequences obtained for each iteration of the number of mismatches allowed for barcode rescue. Solid lines represent the new chimeric sequences, and dashed lines represent the total number of non‐chimeric new reads. Colour indicates library type (black: type A; grey: type B)

Importantly, the number of new reads, excluding chimeric reads, retained during demultiplexing remains higher than the number of new chimeric sequences detected by process_radtags when we increase the number of mismatches for barcode rescue up to three (Figure 6). Past this point, when the number of mismatches equals the distance between barcodes, the number of new chimeric sequences detected overtakes the number of new retained reads, indicating that increasing the number of mismatches past three has no additional benefit.

### 3.4 Quantification of different types of chimeric sequences

**FIGURE 7:**
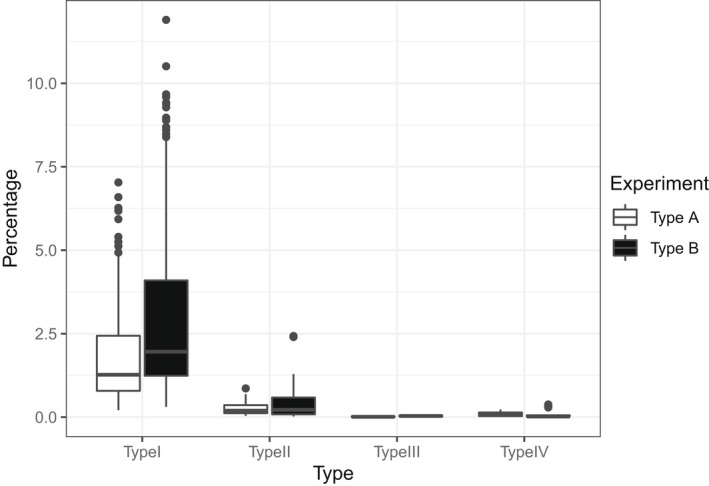
Percentage of chimeric sequences in each library type, relative to the total number of reads per plate sequenced

When the multiplexed groups are equally sized, containing 9 samples, among all possible combinations of inner barcodes (*n* = 81), 11.1% identify genuine reads while the remaining 88.9% identify chimeric sequences. In these cases, in 56 of 75 multiplexed groups, only type I chimeras are detectable. Since type II‐IV chimeras are only detectable in a much smaller fraction of multiplexed groups, type I chimeras seem to be the predominant fraction of chimeras per library type (Figure 7). When only type I chimeras are detectable, type II and III chimeras will be identified as type I chimeras, while type IV chimeras will be misidentified as genuine samples.

**FIGURE 8:**
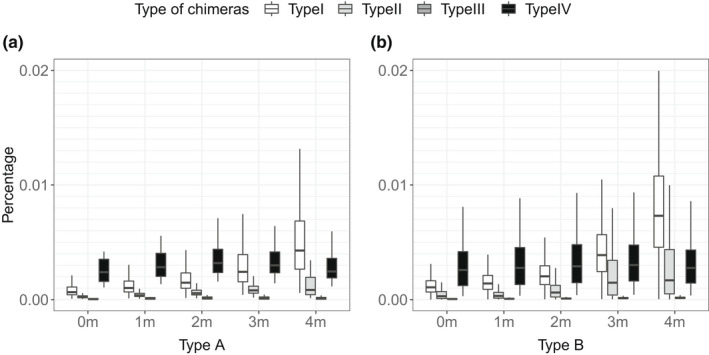
Percentages of four different types of chimeras in each library type. Percentages have been calculated individually for each combination of barcodes included in all multiplexed groups

In multiplexed groups where the number of multiplexed samples was lower than 9, it is possible to identify type II, III and IV chimeras. In these cases, in 19 of the 75 multiplexed groups in our protocol, type IV chimeras are the most abundant chimeras when less than 3 mismatches were allowed for barcode rescue (Figure 8).

As it was only possible to detect type IV chimeras in 19 of the 75 multiplexed groups, we estimated the expected total number of type IV chimeras should the protocol had been constructed to detect all type IV chimeras. In type A libraries, 182,768 type IV chimeras (0.01% of the total reads from the library) were observed, while the estimated number of type IV chimeras is 28,280,095 reads (1.56% of the total reads in the library). In type B libraries, we observed 205,518 type IV chimeras (0.019% of the total number of reads in the library) of the estimated 13,713,202 reads (1.29% of the total number of reads in the library).

## 4 DISCUSSION

Highly multiplexed approaches for reduced‐representation libraries such as RAD‐seq have considerably reduced the cost of genotyping of hundreds of samples (Bayona‐Vásquez et al., 2019; Franchini et al., 2017). However, library preparation methods introduce a number of artefacts that must be considered when designing a RAD‐seq study and its analysis (Andrews et al., 2016). The formation of sequences with chimeric adapters, particularly the ones produced by index hopping, is one of such artefacts, which a number of previous studies have attempted to quantify (Costello et al., 2018; MacConaill et al., 2018; van der Valk et al., 2020).

Here, we extend these analyses to a much larger (total *n* = 639) and highly multiplexed experiment (86 to 100 samples multiplexed), such that has now become common in ecological and evolutionary genomic research. Additionally, we consider variation in the library preparation protocol that affects the proportions of chimeric sequences by analysing chimeric sequences formed during a) sequencing only and b) indexing PCR combined with amplification during sequencing. Finally, we assess the effects of barcode rescue on the proportion of chimeric reads identified and quantify specific types of chimeric sequences (types II–IV) that are impossible to detect in a typical experiment with the same number of samples in every multiplexed group. As our model system *Apodemus* spp. does not have a reference genome available, we were not able to identify intra‐individual chimeras: chimeric sequences produced between different sequences from the same individual. Such sequences can typically be removed from downstream analysis by mapping to a reference genome.

Overall, we show that the proportion of chimeric sequences is generally low for type A libraries: mean = 0.65%, median = 0.59%, stdev = 0.21. Pooling samples early in the protocol (prior to the indexing PCR, as in our type B libraries) approximately doubles the proportion of detectable chimeric sequences: mean = 1.15%, median = 1.09%, stdev = 0.43, thus increasing read misassignment.

We also show that this proportion is relatively stable throughout several sequencing runs (Table S4, Figure 4). In our case, more chimeric sequences identified in library type A‐4 have likely arisen due to the inclusion of degraded DNA samples. As read length negatively correlates with the frequency of index hopping in a sequencing library (van der Valk et al., 2020), it could explain the greater proportion of chimeric reads in type A‐4 library compared to other runs of libraries of type A.

Previous studies (van der Valk et al., 2020) have identified similar percentages of chimeric reads—0.47%—to those obtained in our type A libraries, in a similar protocol that eliminated the possibility of generating chimeras at the indexing PCR stage. In other works, higher proportions of chimeric reads were reported, including in the PCR‐free protocols (1.5% [Ros‐Freixedes et al., 2018]), Illumina Guidelines (2% [Illumina, 2017]) or 1.2% in a study by Costello et al. (2018). The latter study explained their proportions by low yield of the libraries and a high proportion of free‐floating primers on the flow cell.

Our experimental design, with all barcodes separated by 4 nucleotides to minimize read misassignment due to sequencing errors, demonstrated that increasing the number of allowed mismatches during barcode rescue in a demultiplexing step increases the proportion of chimeric sequences by as much as 10‐fold (Figure 3). Our data shows that allowing more than 2 nucleotide difference in barcode rescue results in extremely high proportion of chimeric sequences, to the point where they become more prevalent in the data than the increase in the number of retained reads due to barcode rescue.

Our results indicate that type I chimeras are the most frequent type of chimeras (0.617% and 1.082% of the total reads for libraries of type A and type B, respectively), having in mind that our protocol was dominated by equally sized multiplexed groups. Although the frequency of non‐type I chimeric sequences detected in our protocol is much lower (0.101%, 0.005% and 0.07%, of the total reads for type II, III and IV chimeras in type A libraries; 0.112%, 0.02% and 0.072%, of the total reads for chimeras of types II, III and IV in type B libraries), most chimeras of type IV are undetectable in our protocol. If our protocol allowed for detection of all type IV chimeras, we estimate that they would constitute a 100‐fold larger fraction of chimeric sequences.

Therefore, the principal issue with type IV chimeras is that they are misassigned as genuine samples and they are impossible to detect in the analysis pipeline when multiplexed groups are of the same size. Type II and III chimeras, in contrast, are routinely classified as type I chimeras in RAD‐seq protocols with equally sized multiplexed groups and therefore can be removed during the analysis.

It is difficult to assess the exact impact of chimeric sequences on downstream analyses in our design, as it did not allow the detection of all types of chimeric sequences across all samples. We attempted to estimate the number of SNPs derived from chimeric sequences kept in the pipeline by treating demultiplexed chimeric sequences, classified by the combination of barcodes, demultiplexing group and plate as samples in the Stacks pipeline (Appendix S1: Section 1).

Most of the identified chimeric sequences did not achieve enough coverage to pass through the pipeline. In cases where chimeras did form stacks, the SNPs called were rare and most of them would be filtered out using population filtering options in Stacks (parameters ‐p or ‐r in populations package). When we allowed 0 mismatches for barcode rescue, 379 SNPs were called from chimeric sequences, out of which only 9 remained when the SNPs were required to be present in at least 10 samples. When 2 and 4 barcode mismatches were allowed, 1741 and 54,184 SNPs were called, of which 30 and 239 remained in the pipeline, respectively.

These results suggest that the number of chimeric sequences going into downstream analysis is very low, and their effects on called SNPs are likely to be negligible if the appropriate quality checks in the Stacks pipeline are followed. Factors such as allowing stacks with low coverage, the inability to remove PCR duplicates, low‐quality DNA and/or the use of a high number of mismatches for barcode rescue will increase the number of recruited chimeras, and therefore the number of SNPs derived from chimeric sequences present in downstream analysis. We suggest the following steps that would minimize the impact of the chimeric sequences in any highly multiplexed RAD‐seq experiment.

In experimental designs where costs are less constrained, we would recommend the use of fixed pairs of inner and outer adapters for each sample and multiplexed groups, respectively. Although this increases the cost associated with development and adapter synthesis, fixed pairs of barcodes will minimize the probability of biasing downstream analyses due to read misassignment (van der Valk et al. (2020)). We would also recommend performing indexing PCRs on each sample individually. PCR duplicates might have little effect on genotype calls (Euclide et al., 2020); however, we still recommend the inclusion of a random nucleotide to identify them. The major benefit of being able to identify PCR duplicates and chimeras is the ability to increase the number of PCR cycles in the samples' amplification step, increasing the amount of input material available. In cases where the input materials are scarce and/or degraded, controlling for chimeric sequences becomes more important, as their proportion increases with shorter read length and increasing number of mismatches allowed during barcode rescue.

When costs are a limiting factor, we suggest adopting a hybrid approach, similar to the one described here: use of fixed pairs of inner barcodes only and pooling the samples for the indexing PCR. This approach still enables adequate control of the chimeric sequences in the data, while saving costs during library preparation. Nevertheless when using this approach, one should consider not including all combinations of inner barcodes in every multiplexed group to be able to estimate the frequency of type IV chimeras that are being misassigned to genuine samples.

## CONFLICT OF INTEREST

The authors declare no conflict of interest.

## Supporting information


Appendix S1

## Data Availability

All the code and the demultiplexing information produced by process_radtags, as well as the tables used to perform all the analyses described here, are available on GitHub: https://github.com/Marisa89/chimeric\_adapters. Since the sequencing data that we could deposit in a repository are already demultiplexed and therefore would not allow reproduction of the analyses presented here, we will make raw sequencing data (before demultiplexing) available upon request. All the analyses presented here can be reproduced using the Stacks log files included in the GitHub repository listed above.
