# Supplementary material for: Identification and quantification of chimeric sequencing reads in a highly multiplexed RAD‐seq protocol
Source: Mol Ecol Resour. 2022 Jun 27;22(8):2860–70. doi: 10.1111/1755-0998.13661 (PMC9796921; doi:10.1111/1755-0998.13661)
Supplement: Supplementary file 1 — Appendix S1 [file MEN-22-2860-s001.pdf]

Supplementary Materials: Identification and quantification of chimeric sequencing reads in a highly multiplexed RAD-seq protocol

Maria Luisa Martin Cerezo[1,2*], Rohan Raval [1], Bernardo de Haro Reyes [1], Marek Kucka[3], Frank Yingguang Chan[3] and Jarosław Bryk [1*]

Table 1: i5 adapter's sequences

**Adapter name**	**Sequence**
i5-top_#01_AAGACTGG	CGCTCTTCCGATCTVBBNAAGACTGGTGCA/3Phos/
i5-top_#02_ATGTTGGC	CGCTCTTCCGATCTVBBNATGTTGGCTGCA/3Phos/
i5-top_#04_CCTCATCT	CGCTCTTCCGATCTVBBNCCTCATCTTGCA/3Phos/
i5-top_#05_CGGAATTG	CGCTCTTCCGATCTVBBNCGGAATTGTGCA/3Phos/
i5-top_#06_CAAGGTGA	CGCTCTTCCGATCTVBBNCAAGGTGATGCA/3Phos/
i5-top_#07_GACTTGAG	CGCTCTTCCGATCTVBBNGACTTGAGTGCA/3Phos/
i5-top_#10_TCCTTCAC	CGCTCTTCCGATCTVBBNTCCTTCACTGCA/3Phos/
i5-top_#11_TGTCAGTG	CGCTCTTCCGATCTVBBNTGTCAGTGTGCA/3Phos/
i5-top_#12_TTCTGAGG	CGCTCTTCCGATCTVBBNTTCTGAGGTGCA/3Phos/
i5-bottom_#01_AAGACTGG	/5Phos/CCAGTCTTNVVBAGATCGGAAGAGCGTCGTGTAGGGAAAGAGTGT
i5-bottom_#02_ATGTTGGC	/5Phos/GCCAACATNVVBAGATCGGAAGAGCGTCGTGTAGGGAAAGAGTGT
i5-bottom_#04_CCTCATCT	/5Phos/AGATGAGGNVVBAGATCGGAAGAGCGTCGTGTAGGGAAAGAGTGT
i5-bottom_#05_CGGAATTG	/5Phos/CAATTCCGNVVBAGATCGGAAGAGCGTCGTGTAGGGAAAGAGTGT
i5-bottom_#06_CAAGGTGA	/5Phos/TCACCTTGNVVBAGATCGGAAGAGCGTCGTGTAGGGAAAGAGTGT
i5-bottom_#07_GACTTGAG	/5Phos/CTCAAGTCNVVBAGATCGGAAGAGCGTCGTGTAGGGAAAGAGTGT
i5-bottom_#10_TCCTTCAC	/5Phos/GTGAAGGANVVBAGATCGGAAGAGCGTCGTGTAGGGAAAGAGTGT
i5-bottom_#11_TGTCAGTG	/5Phos/CACTGACANVVBAGATCGGAAGAGCGTCGTGTAGGGAAAGAGTGT
i5-bottom_#12_TTCTGAGG	/5Phos/CCTCAGAANVVBAGATCGGAAGAGCGTCGTGTAGGGAAAGAGTGT

Table 2: i7 adapter's sequences

**Adapter name**	**Sequence**
i7-top_#01_AGAGTTCG	GTGACTGGAGTTCAGACGTGTGCTCTTCCGATCTVBBNAGAGTTCG
i7-top_#02_ACCTGTTG	GTGACTGGAGTTCAGACGTGTGCTCTTCCGATCTVBBNACCTGTTG
i7-top_#03_CTGGTTCA	GTGACTGGAGTTCAGACGTGTGCTCTTCCGATCTVBBNCTGGTTCA
i7-top_#04_CGACAAGA	GTGACTGGAGTTCAGACGTGTGCTCTTCCGATCTVBBNCGACAAGA
i7-top_#05_CAGTCGAA	GTGACTGGAGTTCAGACGTGTGCTCTTCCGATCTVBBNCAGTCGAA
i7-top_#06_GTCAGAAC	GTGACTGGAGTTCAGACGTGTGCTCTTCCGATCTVBBNGTCAGAAC
i7-top_#7_TTGTTCCG	GTGACTGGAGTTCAGACGTGTGCTCTTCCGATCTVBBNTTGTTCCG
i7-top_#8_TCGCATTC	GTGACTGGAGTTCAGACGTGTGCTCTTCCGATCTVBBNTCGCATTC
i7-top_#9_TCGAACCA	GTGACTGGAGTTCAGACGTGTGCTCTTCCGATCTVBBNTCGAACCA
i7-bottom_#01_AGAGTTCG	TACGAACTCTNVVBAGATCGGAAGAGCA
i7-bottom_#02_ACCTGTTG	TACAACAGGTNVVBAGATCGGAAGAGCA
i7-bottom_#03_CTGGTTCA	TATGAACCAGNVVBAGATCGGAAGAGCA
i7-bottom_#04_CGACAAGA	TATCTTGTCGNVVBAGATCGGAAGAGCA
i7-bottom_#05_CAGTCGAA	TATTCGACTGNVVBAGATCGGAAGAGCA
i7-bottom_#06_GTCAGAAC	TAGTTCTGACNVVBAGATCGGAAGAGCA
i7-bottom_#7_TTGTTCCG	TACGGAACAANVVBAGATCGGAAGAGCA
i7-bottom_#8_TCGCATTC	TAGAATGCGANVVBAGATCGGAAGAGCA
i7-bottom_#9_TCGAACCA	TATGGTTCGANVVBAGATCGGAAGAGCA

Table 3: Combinatorial outer adapter sequences

**Adapter name**	**Sequence**
i501_AGCATGGA	AATGATACGGCGACCACCGAGATCTACAC{AGCATGGA}ACACTCTTTCCCTACACGAC*G
i502_CCTGGAAT	AATGATACGGCGACCACCGAGATCTACAC{CCTGGAAT}ACACTCTTTCCCTACACGAC*G
i503_GCAAGCAA	AATGATACGGCGACCACCGAGATCTACAC{GCAAGCAA}ACACTCTTTCCCTACACGAC*G
i504_TGAGGATG	AATGATACGGCGACCACCGAGATCTACAC{TGAGGATG}ACACTCTTTCCCTACACGAC*G
i701_ACACTCAG	CAAGCAGAAGACGGCATACGAGAT{CTGAGTGT}GTGACTGGAGTTCAGACGTGTGC*T
i702_CAGTCGAA	CAAGCAGAAGACGGCATACGAGAT{TTCGACTG}GTGACTGGAGTTCAGACGTGTGC*T
i703_GGCTCAAT	CAAGCAGAAGACGGCATACGAGAT{ATTGAGCC}GTGACTGGAGTTCAGACGTGTGC*T
i704_TTCCGCTT	CAAGCAGAAGACGGCATACGAGAT{AAGCGGAA}GTGACTGGAGTTCAGACGTGTGC*T

Table 4: Proportion of chimeric sequences per plate. Median (+/- standard deviation), mean, maximum and minimim values are shown.

Plate	Mismatches	Median	Stdev	Mean	Max	Min
PlateA-1	0.00	0.54	0.14	0.55	0.87	0.36
PlateA-2	0.00	0.59	0.17	0.64	0.91	0.38
PlateA-3	0.00	0.49	0.12	0.53	0.69	0.33
PlateA-4	0.00	0.90	0.23	0.89	1.21	0.44
PLateB-1	0.00	0.83	0.19	0.89	1.39	0.68
PlateB-2	0.00	1.32	0.16	1.30	1.51	1.03
PLateB-3	0.00	1.25	0.67	1.28	2.33	0.31

Analysis were performed using the four type A libraries. Demultiplexed chimeras, classified by the combination of barcodes, demultiplexing group and plate, were used as samples in Stacks (ustacks, cstacks, sstacks, tsv2bam, gstacks). SNPs were called without filtering, with -r set up to 10. Analysis were run for 0, 2 and 4 mismatches.
